# Overexpression of soybean *GmNAC19* and *GmGRAB1* enhances root growth and water-deficit stress tolerance in soybean

**DOI:** 10.3389/fpls.2023.1186292

**Published:** 2023-05-31

**Authors:** Mitra Mazarei, Pratyush Routray, Sarbottam Piya, C. Neal Stewart, Tarek Hewezi

**Affiliations:** ^1^ Department of Plant Sciences, University of Tennessee, Knoxville, TN, United States; ^2^ Center for Agricultural Synthetic Biology, University of Tennessee, Knoxville, TN, United States

**Keywords:** soybean, GmNAC19, GmGRAB1, GmTUBBY, overexpression, root growth, seed yield, water deficit

## Abstract

Soybean (*Glycine max*) is an important crop in agricultural production where water shortage limits yields in soybean. Root system plays important roles in water-limited environments, but the underlying mechanisms are largely unknown. In our previous study, we produced a RNA-seq dataset generated from roots of soybean at three different growth stages (20-, 30-, and 44-day-old plants). In the present study, we performed a transcriptome analysis of the RNA-seq data to select candidate genes with probable association with root growth and development. Candidate genes were functionally examined in soybean by overexpression of individual genes using intact soybean composite plants with transgenic hairy roots. Root growth and biomass in the transgenic composite plants were significantly increased by overexpression of the *GmNAC19* and *GmGRAB1* transcriptional factors, showing up to 1.8-fold increase in root length and/or 1.7-fold increase in root fresh/dry weight. Furthermore, greenhouse-grown transgenic composite plants had significantly higher seed yield by about 2-fold than control plants. Expression profiling in different developmental stages and tissues showed that *GmNAC19* and *GmGRAB1* were most highly expressed in roots, displaying a distinct root-preferential expression. Moreover, we found that under water-deficit conditions, overexpression of *GmNAC19* enhanced water stress tolerance in transgenic composite plants. Taken together, these results provide further insights into the agricultural potential of these genes for development of soybean cultivars with improved root growth and enhanced tolerance to water-deficit conditions.

## Introduction

Plant root systems exhibits diversity in morphology, physiology, and architecture, which are collectively impacted by variable and unpredictable soil environment. Root uptake of belowground resources, such as water and nutrients, are fundamental to plant productivity. Roots play important roles in water-limited environments as robust root growth could enhance crop production in water-deficient soil. While roots are underground, unseen, and relatively unappreciated, they represent the foundation for crop yield (Franco et al., 2011; Schmidt, 2014; Rellán-Álvarez et al., 2016; Lynch, 2019; Maurel and Nacry, 2020).

Soybean (*Glycine max*) is one of the most important crops globally. However, soybean production is often impacted by various biotic and abiotic stress factors. Among them, water shortage is an important limiter for soybean yield, which is especially important during pod filling stage (Frederick et al., 2001; Brevedan and Egli, 2003; Liu et al., 2003; Manavalan et al., 2009; Xionga et al., 2021). Advanced technologies have yielded information for development of drought-tolerant soybean cultivars, either through molecular breeding or genetic engineering approaches (Arya et al., 2021; Saleem et al., 2022).

Several studies have used bioinformatic tools such as differential gene expression analysis and homologous sequence similarity to the genes with known function in rice and Arabidopsis to identify candidate genes for root growth and water-deficit tolerance in soybean. These systematic-based analyses detected transcriptional factors of the MYB, bHLH, AP2-EREBP, NAC, WRKY, bZIP, and C2H2-zinc finger gene families as prime factors (Le et al., 2011; Le et al., 2012; Neves-Borges et al., 2012; Chai et al., 2015; Ha et al., 2015; Song et al., 2016; Hussain et al., 2017; Melo et al., 2018; Zhou et al., 2020; Shahriari et al., 2022; Xuan et al., 2022). However, the majority of these studies lack the functional characterization of these candidate genes. To date, the molecular functions of a limited member of the transcription factors have been shown in soybean (Chen et al., 2007; Zhang et al., 2009; Gao et al., 2011; Hao et al., 2011; Quach et al., 2014; Fuganti-Pagliarini et al., 2017; Ning et al., 2017; Shi et al., 2018; Nguyen et al., 2019; Wei et al., 2019; Yang et al., 2019; Yang et al., 2020; Wang et al., 2020; Wang et al., 2021; Chen et al., 2021; Leng et al., 2021; Yuan et al., 2021).

The objective of this study was to identify candidate genes with special reference to root growth and water-deficit tolerance in soybean. We (i) used transcriptome analysis of the RNA-seq data, (ii) identified the candidate genes, and (iii) examined the functionality by overexpressing individual genes in transgenic composite soybean plants. Our results provide important insights into the potential applications of these genes for line development with desire traits in this economically important crop.

## Materials and methods

### Selection of candidate genes

We previously produced RNA-seq data from roots of soybean (‘Williams 82’) at three different growth stages (20-, 30-, and 44-day-old plants) with three biological replicates for each time point as described in Niyikiza et al. (2020). In the present study, we performed a transcriptome analysis of the RNA-seq data generated in Niyikiza et al. (2020). Genes with the highest expression levels at each of the three growth stages were identified. Then, genes showing high expression across the three growth stages were selected and screened through translational literature for information about genes that associate with root growth in plants.

### Expression patterns of candidate genes

For expression profiling of the candidate genes, the publicly available RNA-seq datasets were used. Description and accession number of these RNA-seq datasets were provided in Piya et al. (2023). Heatmap plot was created by the PhytoMine tool (https://phytozome.jgi.doe.gov) presented at the Phytozome v12 website (https://phytozome.jgi.doe.gov). Gene expression analysis and heatmap plot construction were performed as described in Piya et al. (2023).

### Isolation of open reading frame (ORF) of candidate root genes

Total RNA was isolated from root tissue of ‘Williams 82’ soybean using the RNeasy Plant Mini Kit (Qiagen, Valencia, CA, USA). Then, first-strand cDNA was synthesized using High-Capacity cDNA Reverse Transcription kit (Applied Biosystems, Foster City, CA, USA). The ORF corresponding to each target gene were amplified via PCR using gene-specific primers. Primers were designed to create *Asc*I (at the 5’ end) and *Avr*II or *Bam*HI (at the 3’ end) restriction sites (**Supplementary Table S1**). The individual PCR products were cloned into the pGEM vector (Promega, Madison, WI, USA) for sequence confirmation.

### Construction of the soybean transformation vector

Sequence-confirmed ORF fragments were subcloned into the binary vector pG2RNAi2 (GenBank KT954097) by replacing the GUS linker with each target gene under the control of the soybean ubiquitin (*GmUbi*) promoter and the RuBisCO small subunit (*rbc*S) terminator. The binary vector contains the green fluorescence protein (GFP) reporter under the control of the *Cauliflower mosaic virus* (CaMV 35S) promoter and the nopaline synthase (*NOS*) terminator as selective marker for transgenic hairy roots.

### Generation of transgenic soybean hairy roots

*Agrobacterium rhizogenes* strain K599 was used for the generation of the soybean hairy roots as previously described (Rambani et al., 2020a; Rambani et al., 2020b; Piya et al., 2022). The individual target genes cloned into the pG2RNAi2 binary vector and the empty pG2RNAi2 vector were transferred into *A. rhizogenes* by the freeze-thaw method. Transgenic composite hairy root plants were generated by injecting *A*. *rhizogenes* containing different binary constructs in the hypocotyl of 5-day-old ‘Williams 82’ seedlings. Transgenic composite hairy root plants expressing the empty vector were also generated and used as negative controls. About 25 transgenic soybean hairy root lines for each construct were generated. The inoculated soybean seedlings were maintained in growth chambers (Percival Scientific Inc. Perry, IA, USA) at 26°C under a photoperiod of 16 h light/8 h dark cycle with 150 µmol/m^2^ s light intensity. Approximately, four to six weeks after agroinoculation, transgenic hairy roots were detected based on GFP expression, using an epifluorescent microscope model SZX12 (Olympus America, Center Valley, PA, USA) with a GFP filter set at 487/509 nm excitation/emission wavelengths. The tap root and GFP-negative hairy roots were excised.

### Growth characteristics and water-deficit treatment

The soybean composite plants with the GFP-positive hairy roots were grown in 0.6 L pots containing potting mix (Sun Gro Horticulture, Agawam, MA, USA) under growth chamber conditions mentioned above. For hairy root growth under no stress, maximum root length and root fresh/dry biomass of the soybean composite plants with the GFP-positive hairy roots were measured by ruler and weighing scale, respectively. For yield evaluation under no stress, the soybean composite plants with the GFP-positive hairy roots were transplanted into 4-liter pots containing potting mix and grown in a greenhouse (photoperiod of 16 h light/8 h dark cycle and 25 °C temperature with fluctuations from a minimum of 22 °C to a maximum of 28 °C) to full maturity. The pod number, seed count, and total seed weight per plant were measured. To assess the effects of water-deficit stress, an experiment was performed in 0.6 L pots (potting mix) in a growth chamber. Water deficit treatment on composite plants with 10 GFP-positive hairy roots was applied by withholding water for four weeks after plants were allowed to acclimate for one-week in pots. The effect of water deficit stress was assessed by monitoring wilting and survival of the plants.

### Statistical analysis

Means were analyzed in SAS version 9.4 (SAS Institute Inc., Cary, NC, USA) using one-way ANOVA using Fisher’s least significant difference method. Differences were considered statistically significant at *P* ≤ 0.05.

## Results

### Selection of candidate genes

**Figure 1:**
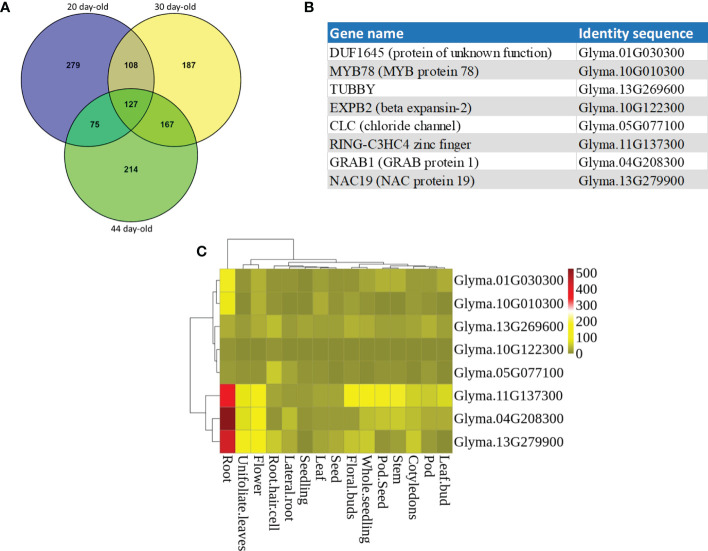
Candidate genes selected from transcriptome analysis of the RNA-seq data generated in Niyikiza et al. (2020). **(A)** Venn diagrams showing unique and shared high expressed genes in the roots of soybean at 20, 30, and 44 days of growth stages. **(B)** The identity of the eight candidate genes. **(C)** Expression profiling of the candidate genes using the publicly available RNA-seq datasets. Heatmap expression profiles of candidate genes in different tissues of soybean. The abundance of each transcript is represented by the color bar. Red indicates higher and green indicates lower expression levels.

A transcriptome analysis of the RNA-seq data which potentially included the entire expression profile of the soybean root genome at three different growth stages (20, 30, and 44 day-old) was performed. We first selected the top 600 genes from each of the three growth stages with the most expression levels (Log_2_ RPKM of 10.81 to 6.71) (Niyikiza et al., 2020). Venn diagrams for the top 600 genes were constructed that showed 127 genes were common to the three growth stages (**Figure 1A**). Several of these common genes (**Supplementary Table S2**) corresponded to genes with function in root growth/development and/or tolerance to stress response. A total of eight genes from the common gene list were selected as candidate genes (**Figure 1B**). Furthermore, *in silico* expression profile of these candidate genes in different developmental stages and tissues showed that the expression patterns of these genes were varied among different organs and tissues (**Figure 1C**). *NAC19*, *GRAB1*, and *Ring zinc finger* were most expressed in roots, displaying a distinct root-preferential expression pattern. Expression of *MYB78* and *DUF1645* was higher in roots among all the tissues. *TUBBY* and *CLC* had higher expression in root hair compared to a low expression level in other tissues. *EXPB2* had very low expression level in all the tissues.

### Initial screening of the candidate genes in soybean

Initially, soybean composite plants overexpressing the individual *CLC, EXPB2, RING-C3HC4 zinc finger, MYB78, DUF1645, NAC19, TUBBY*, and *GRAB1* genes (**Figure 1B**) were generated. About 85% of the hairy roots were transgenic exhibiting GFP fluorescence. Four weeks after agroinoculation, the tap root and GFP-negative hairy roots were eliminated (**Supplemental Figure S1**). Six independent soybean composite plants per each construct with 10 GFP-positive hairy roots were used for water-deficit tolerance assays. The plants were grown in potting mix supplemented with water for one week in the growth chamber before the onset of the water-deficit stress. Then, the plants were left in the growth chamber without water. After three weeks of withholding water, wilting of the plants was visible. However, after four weeks of withholding water, only transgenic composite plants transformed with *NAC19, TUBBY*, and *GRAB1* genes survived (**Supplemental Figure S2**).

### Selection of top performing candidate genes

Based on the initial results (**Supplemental Figure S2**), we selected *NAC19*, *TUBBY*, and *GRAB1* genes for further experiments. New sets of soybean composite plants overexpressing the individual *NAC19*, *TUBBY*, and *GRAB1* genes were generated. Visual estimation showed that about 90% of the hairy roots were transgenic by exhibiting the GFP fluorescence.

### Non-stress condition

**Figure 2:**
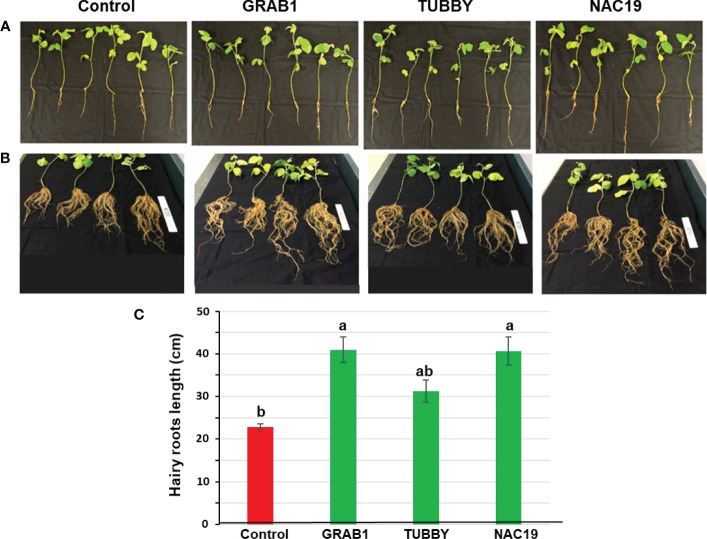
Growth characteristics of transgenic soybean hairy roots overexpressing the candidate genes under non-stress conditions. **(A)** Representatives of transgenic hairy roots at five weeks after agroinoculation. The tap root and GFP-negative hairy roots were excised. **(B)** Representatives of transgenic hairy roots from **(A)** grown for an additional three weeks. **(C)** Average hairy roots lengths obtained from **(B)** composite plants. Bars represent mean values of eight biological replicates (composite plants) ± standard error. Bars with different letters are significantly different at *P* ≤ 0.05 as tested by one-way analysis of variance followed by a Fisher’s least significant difference. Scale ruler = 15 cm.

**Figure 3:**
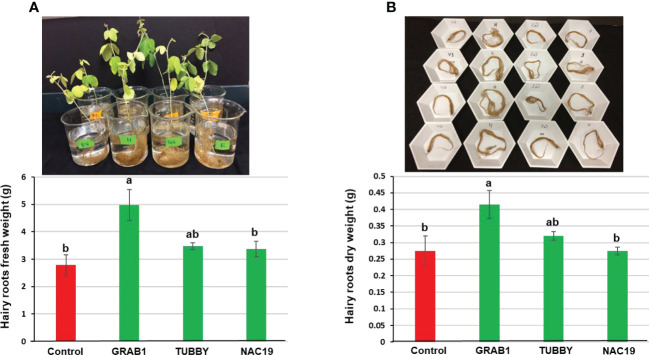
Growth characteristics of transgenic soybean hairy roots overexpressing the candidate genes under non-stress conditions. **(A)** Representatives of hairy roots fresh weight and **(B)** dry weight at eight weeks after agroinoculation. Bars represent mean values of eight biological replicates (composite plants) ± standard error. Bars with different letters are significantly different at *P* ≤ 0.05 as tested by one-way analysis of variance followed by a Fisher’s least significant difference.

For evaluation of root growth under non-stress condition, the soybean composite plants at four weeks after agroinoculation (**Figure 2A**) were grown for an additional three weeks. Eight independent soybean composite plants per each construct were used. There were apparent morphological differences between transgenic hairy roots transformed with *NAC19* or *GRAB1* and the control plants (**Figure 2B**). Hairy roots of *NAC19* or *GRAB1* plants were 1.8-fold longer compared to the controls (**Figure 2C**). Moreover, the fresh/dry weights of hairy roots of *GRAB1* plants were increased by up to 1.7-fold compared to the controls (**Figure 3**).

### Agronomic traits evaluation under greenhouse conditions

**Figure 4:**
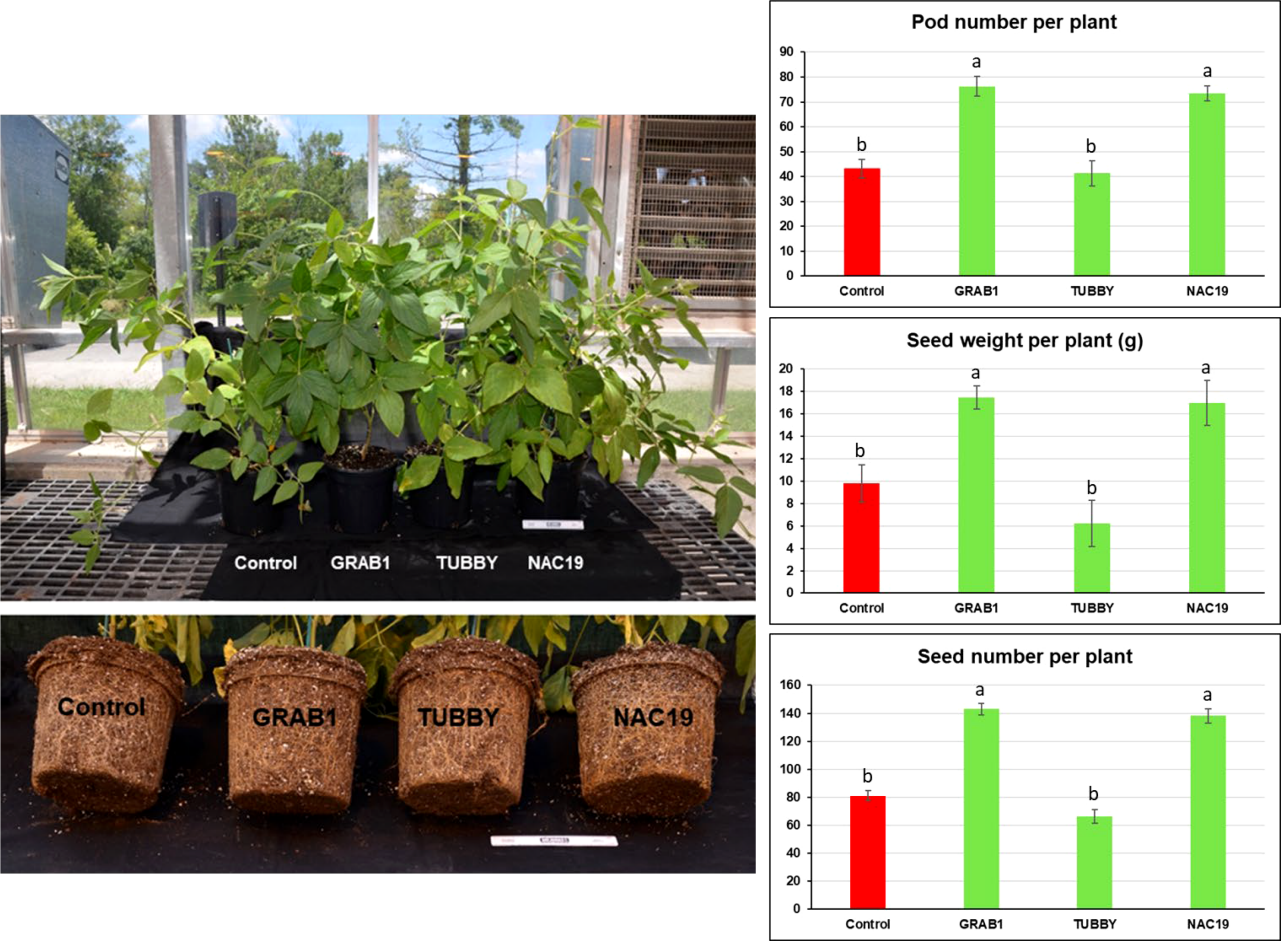
Agronomic trait evaluation of transgenic soybean hairy roots overexpressing the candidate genes under non-stress conditions. The composite soybean plants were grown under greenhouse condition to full maturity. Bars represent mean values of six biological replicates (composite plants) ± standard error. Bars with different letters are significantly different at *P* ≤ 0.05 as tested by one-way analysis of variance followed by a Fisher’s least significant difference. Scale ruler = 15 cm.

For evaluation of seed production, six independent soybean composite plants per construct were grown in the greenhouse to full maturity (**Figure 4**). No visible morphological differences were observed among the plants. The plants with transgenic hairy roots transformed with *NAC19* or *GRAB1* gene produced more pods (up to 1.8-fold), seeds (1.8-fold), and seed weight (1.9-fold) per plant relative to controls. But, there was no significant difference in the single seed weight compared to controls (**Figure 4**).

### Water-deficit stress condition

**Figure 5:**
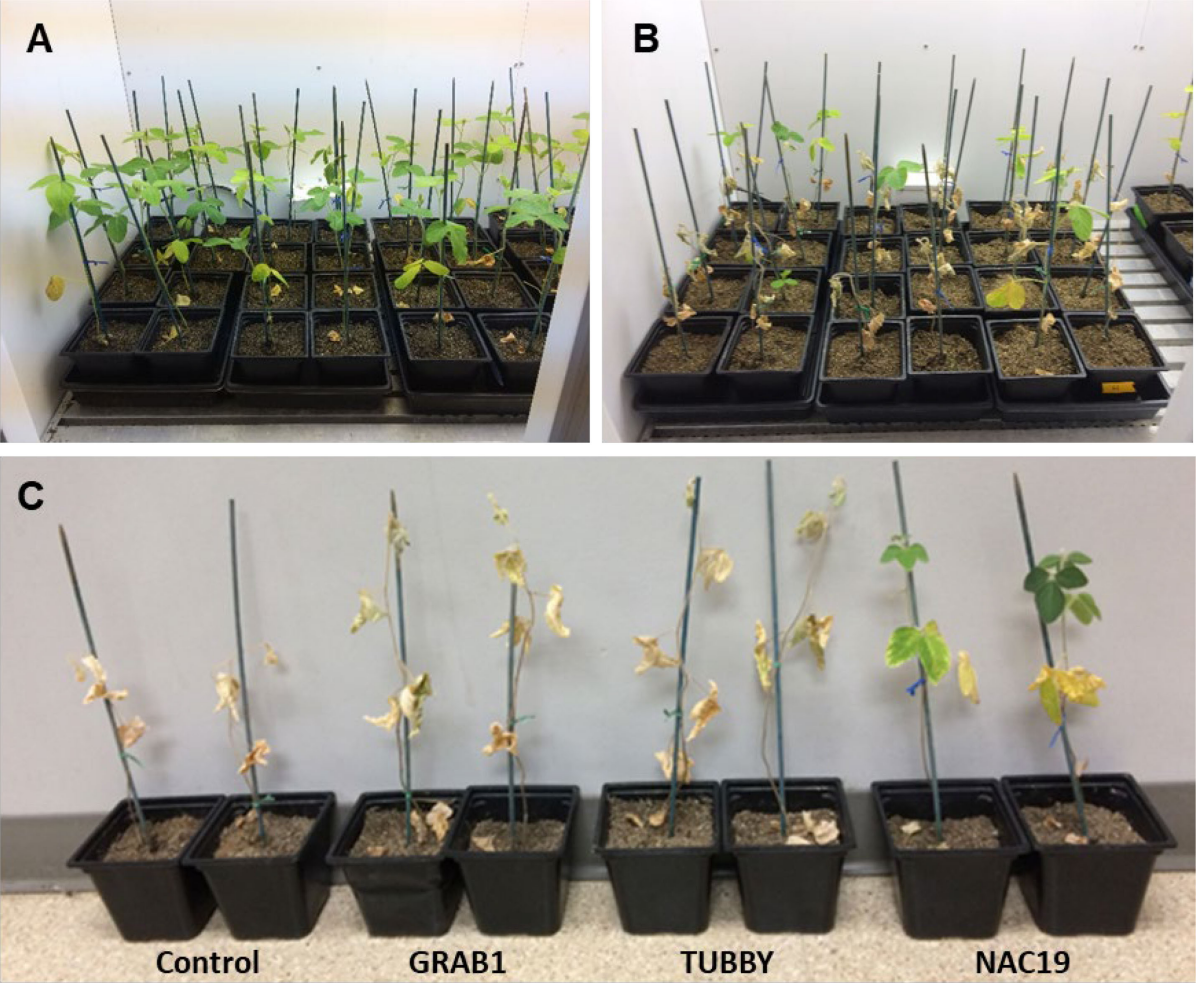
Soybean composite plants with transgenic hairy roots overexpressing the candidate genes under water-deficit stress conditions. **(A)** Representatives of composite plants before subjecting to water-deficit condition. **(B, C)** Representatives of composite plants subjected to water-deficit condition via water deprivation for four weeks.

For evaluation of water-deficit stress tolerance, the soybean composite plants at four weeks after agroinoculation were used. Six independent soybean composite plants per construct with 10 GFP-positive roots were used. The conditions for the plant growth and water-stress treatment were as described above. After four weeks of withholding water, only transgenic composite plants overexpressing *NAC19* gene survived (**Figure 5**).

## Discussion

Water-deficit stress is a major abiotic factor that decreases soybean growth and production (Xionga et al., 2021). Several studies involving genetic mechanism of drought tolerance in soybean have been conducted, but more research is needed to explore and understand the genetic control of drought tolerant traits in soybean (Arya et al., 2021). In this study, we performed a comprehensive transcriptomic analysis of expressed genes in soybean roots at different growth stages to identify genes with probable association with growth and stress tolerance. Our findings provide additional information for the elucidation of the growth and stress responses in soybean.

Our RNA-seq analysis identified *CLC* and *EXPB2* as candidate genes, which have been shown to be involved in root elongation and stress responses in soybean (Guo et al., 2011; Li et al., 2015; Wei et al., 2016). It also identified members of various transcription family members including zinc fingers, MYBs, DUFs, and NACs, which are considered as the prime transcription factors associated with root growth and stress response in soybean (Chai et al., 2015). These findings further validate our RNA-seq approach for the identification of candidate genes.

Yet, functional characterization of relevant genes is often missing in gene discovery, which is absolutely required for crop improvement. Many systematic studies have identified a number of candidate genes for growth and water stress response, providing useful genetic resources for functional analyses and future development of improved soybean (Le et al., 2011; Le et al., 2012; Neves-Borges et al., 2012; Chai et al., 2015; Ha et al., 2015; Song et al., 2016; Hussain et al., 2017; Melo et al., 2018; Zhou et al., 2020; Shahriari et al., 2022; Xuan et al., 2022). However, the functionality of majority of these candidate genes is yet to be elucidated. For example, the NAC gene family known for their involvement in plant growth and stress responses is one of the most studied transcription factors in soybean. As such, numerous NAC candidate genes for growth and water-stress tolerance were identified in soybean (Le et al., 2011; Ha et al., 2015; Hussain et al., 2017; Melo et al., 2018). Nevertheless, to date, the functions of only a few NAC family members have been elucidated in soybean (Hao et al., 2011; Quach et al., 2014; Yang et al., 2019; Yang et al., 2020). Thus, more functional studies of *GmNAC* genes are needed to explore their specific and redundant roles in stress tolerance in soybean. We showed that overexpressing *GmNAC19* using transgenic hairy root system significantly increased root growth under non-stress and enhanced tolerance under water-deficit conditions. Yet, *GmNAC19* overexpression resulted in increased root length, but not root biomass weight. The number and length of the roots have been considered as important factors for root system architecture and higher root biomass weight is thought to be associated with improved lateral root system (Lynch, 2019; Maurel and Nacry, 2020). This may suggest that *GmNAC19* is mainly associated with deep root growth, rather than shallow root growth trait. Furthermore, the *GmNAC19*-overexpressing lines produced higher seed yield than controls grown to full maturity in the greenhouse, a finding that may suggest a positive impact of the increased root growth on seed production. Altogether, our functional characterization of *GmNAC19* provides additional knowledge about potential applications of *GmNAC* genes for development of improved soybeans.

Likewise, overexpression of *GmGRAB1* led to a significant increase in root growth and seed yield under non-stress conditions. However, under water-deficit conditions, *GmGRAB1*-overexpressing lines did not exhibit enhanced stress tolerance. GRAB proteins are novel members of the NAC transcription factor (Xie et al., 1999). The *GmGRAB1* identified in our study is a homolog of rice *OsNAC9*, a *NAC* gene whose overexpression in rice increased grain production under normal conditions and enhanced stress tolerance under water-deficit conditions (Redillas et al., 2012). Taken together, the present study provides further insights into the possible conserved functionality of these homologous *NAC* genes across plant species.

Additionally, our expression analysis indicated that both *GmNAC19* and *GmGRAB1* are primarily expressed in roots, displaying a distinct root-preferential expression. These findings further suggest that *GmNAC19* and *GmGRAB1* may function in roots. It also points out to the potential biotechnological use of their promoters to direct gene expression to the targeted root tissues (Liu and Stewart, 2016).

Our functional characterization involving overexpression of the candidate gene *GmTUBBY* resulted in inconsistent findings. Initial screening of our candidate genes led to selection of *GmTUBBY* along with *GmNAC19* and *GmGRAB1* as potential genes for enhancing water-deficit tolerance. However, further in-depth functional analyses showed no significant changes in root growth or water-deficit stress tolerance in *GmTUBBY*-overexpressing lines. TUBBY-like proteins (TLPs) are transcription factors that have been shown to play important roles in plant growth and development and in responses to biotic and abiotic stresses, including drought tolerance (Bhushan et al., 2007; Chen et al., 2016; Xu et al., 2019; Li et al., 2021). However, there is not much published information about TLPs and their association with abiotic stress responses in soybean. Using bioinformatic tools, a recent study identified 22 TLP genes in soybean genome (Xu et al., 2022). The role of *GmTLP8* in abiotic responses was further investigated by overexpression of *GmTLP8* in soybean showing enhanced tolerance to drought and salt stresses (Xu et al., 2022). These findings provided new insights into the function of TLPs in abiotic stress responses in soybean. Based on the information reported in Xu et al. (2022), the *GmTUBBY* gene identified in our study corresponds to *GmTLP13* whose function in soybean remains to be elucidated. Furthermore, using publicly available gene expression data, the *GmTLP13* was found to be upregulated under drought stress (Xu et al., 2022). The *GmTLP13* upregulation in response to drought stress was further confirmed by qRT-PCR experiments (Xu et al., 2022). Taken together, our present study may also provide additional clues into the function of the TLPs in stress responses in soybean. Further research on the TLP genes enhance understanding of the factors associated with stress tolerance in soybean.

In conclusion, we have shown that overexpression of *GmNAC19* and *GmGRAB1* can enhance root growth and/or tolerance to water-deficit stress in soybean. Additionally, *GmNAC19* and *GmGRAB1* likely function in roots, consistent with their root preferential expression patterns. These results provide further insights into the potential applications of these genes for development of improved soybean cultivars. Future studies of the *GmNAC19* and *GmGRAB1* in stable transgenic soybean with subsequent evaluations under field conditions will further elucidate their functionality in practical agricultural setting. Also, further research on *GmNAC19* and *GmGRAB1* for discovery of downstream target genes would lead to the identification of suit of genes that may act in concert for development of improved soybeans. These studies are expected to yield insights into the mechanisms involved in root growth and drought tolerance and provide information on potential strategies for developing improved soybeans. Our study provides insights for a more rigorous exploration of the role of *GmNAC19* and *GmGRAB1*, which may serve to engineer crops for higher productivity and sustainability.

## Data availability statement

The original contributions presented in the study are included in the article/**Supplementary Material**. Further inquiries can be directed to the corresponding authors.

## Author contributions

MM analyzed RNA-seq data, selected candidate genes, participated in developing the experimental design, performed growth and water-stress experiments, performed data analysis/interpretation, produced figures, and drafted the manuscript. PR cloned the candidate genes, generated the overexpression constructs, performed hairy roots transformation, participated in developing the experimental design, and performed the growth and initial water-stress experiments. SP produced RNA-seq data, and performed bioinformatics analyses. CNS co-conceived the study, participated in designing the study, and participated in the interpretation of results. TH co-conceived the study, participated in designing the study, and participated in data interpretation. All authors participated in contributing to text and the content of the manuscript, including revisions and edits. All authors contributed to the article and approved the submitted version.
