# Supplementary material for: Overexpression of soybean GmNAC19 and GmGRAB1 enhances root growth and water-deficit stress tolerance in soybean
Source: Front Plant Sci. 2023 May 31;14:1186292. doi: 10.3389/fpls.2023.1186292 (PMC10264791; doi:10.3389/fpls.2023.1186292)
Supplement: Supplementary file 1 [file DataSheet_1.pdf]

Supplementary data

Overexpression of soybean *GmNAC19* and *GmGRAB1* enhances root growth and water stress tolerance in soybean

Mitra Mazarei[1,2], Pratyush Routray[1], Sarbottam Piya[1], C. Neal Stewart Jr.[1,2*], Tarek Hewezi[1*]

[1]*Department of Plant Sciences, University of Tennessee, Knoxville, Tennessee, USA*

[2]*Center for Agricultural Synthetic Biology, University of Tennessee, Knoxville, Tennessee, USA*

[*]**Correspondence:** C. Neal Stewart, Jr. and Tarek Hewezi

Emails: nealstewart@utk.edu (CNS); thewezi@utk.edu (TH)

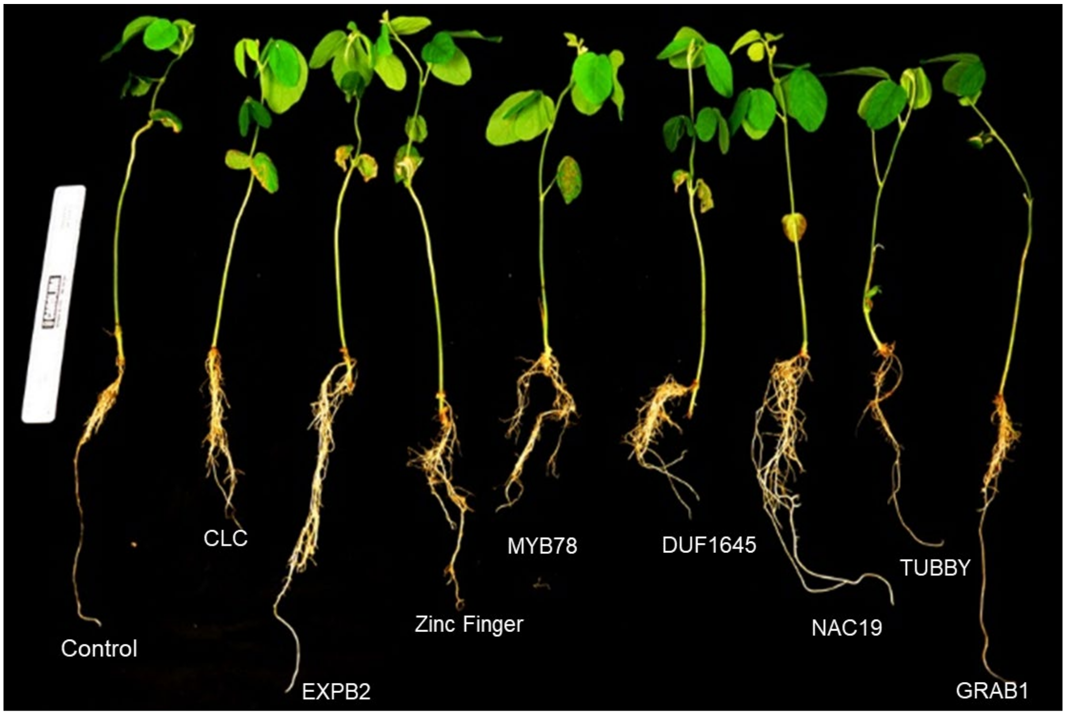

Supplementary Figure S1. Growth characteristics of transgenic soybean hairy roots overexpressing the candidate genes under non-stress conditions. Representatives of transgenic hairy roots at four weeks after agroinoculation. The tap root and GFP-negative hairy roots were excised. Scale ruler = 15 cm.

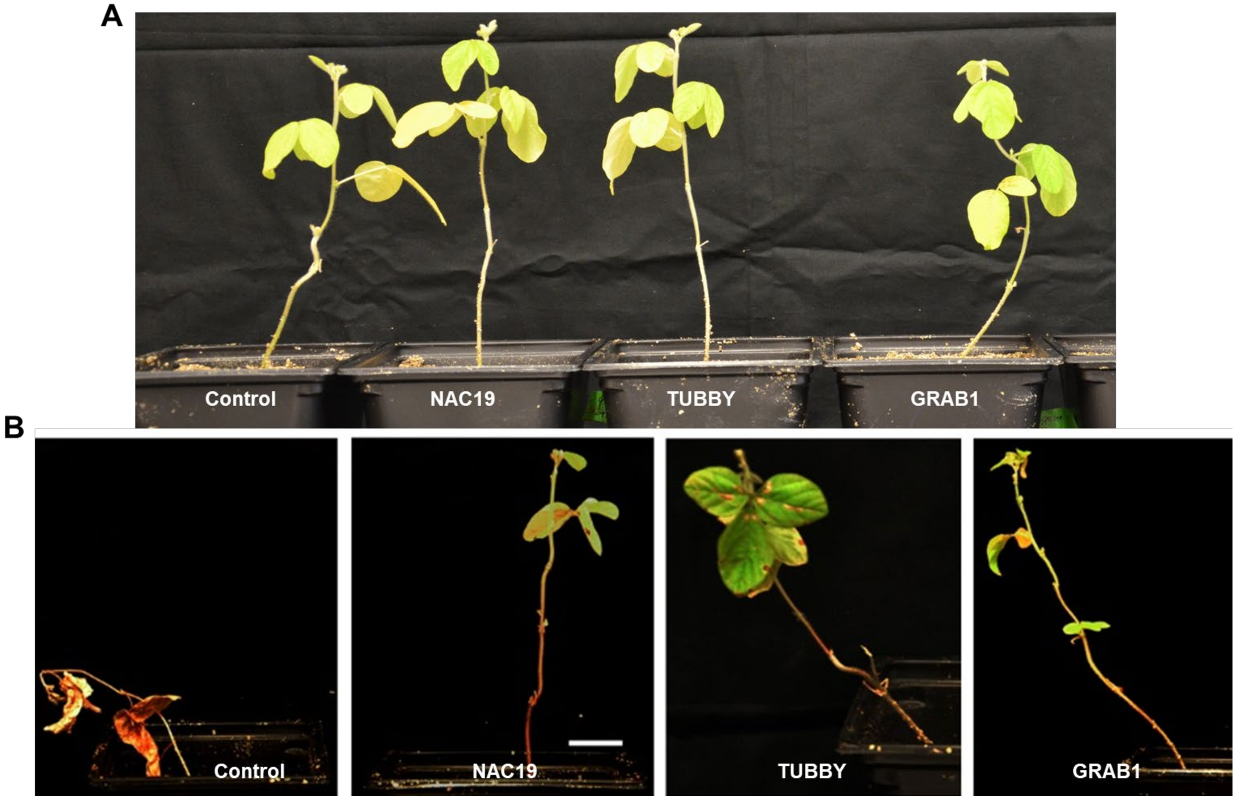

Supplementary Figure S2. Soybean composite plants with transgenic hairy roots overexpressing the candidate genes under water-deficit stress conditions. **(A)** Representatives of composite plants before subjecting to dehydration condition. **(B)** Representatives of composite plants subjected to dehydration condition via water deprivation for four weeks.

Supplementary Table S1. List of primers used in vector construction

Restriction enzyme sites are underlined.

Cloning gene	Primer
Glyma.05G077100 (CLC)	F- GCGGCGCGCCATGGGTGAGGAATCCAGTTTGCTTAAAGAAAG
	R- GCCCTAGGTCACTTCCTCTTTGATTTTGCCAGGTGAG
Glyma.10G122300 (EXPB2)	F- GCGGCGCGCCATGGCTCCTACACTTCAACGTGCACTTTC
	R-GCCCTAGGTTAAAAATTGACAATTGATCTATAAGTCTGATCAATAATCCACCCAGC
Glyma.11G137300 (RING zinc finger)	F- GCGGCGCGCCATGAATTTGGAAACGCAACCAGGAACG
	R- GCGGATCCTTAATCATCCATAACCCAAGTCCTGCTATGTC
Glyma.10G010300 (MYB78)	F- GCGGCGCGCCATGGATGTTAAGAAAGGTGGGTCTGTAG
	R- GCCCTAGGTTATTTCATTTGGAGGTCATAAGAAAGCTGTTGCAAAAAG
Glyma.01G030300 (DUF1645)	F- GAGCCGGCGCGCCATGCAAGCCGTTTCGCTC
	R- GAGCCCCTAGGTCAATAAGGATGAACATTTCTGCTCAATCC
Glyma.13G279900 (NAC19)	F- GAGCCGGCGCGCCATGGGAGTTCCAGAGAAAGACCCTC
	R- GAGCCCCTAGGTCAATTTCTGAACCCGAACCCGACC
Glyma.13G269600 (TUBBY)	F- GAGCCGGCGCGCCATGTCGCTGAGGAAGGTCTTCC
	R- GAGCCCCTAGGTTACTCACAAGCAAGTTTAGTGCCAAAGC
Glyma.04G208300 (GRAB1)	F- GAGCCGGCGCGCCATGAAGGGAGAATTAGAGTTGCCACC
	R- GAGCCCCTAGGTCACATCTTCTGTAGGTACATGAACATGTCC

Supplemental Table S2: Common genes in the roots of soybean at 20, 30, and 44 days of growth stages.

Identity sequence	Gene name
Glyma.10G207000	
Glyma.17G128900	EF-HAND CALCIUM-BINDING DOMAIN CONTAINING PROTEIN
Glyma.20G141600	Ubiquitin and ubiquitinlike proteins // Ubiquitin
Glyma.02G260800	
Glyma.04G118700	Pectinesterase / Pectin methylesterase
Glyma.11G234300	Wound-induced protein WI12 (WI12)
Glyma.08G153600	
Glyma.04G009400	Dehydrin (Dehydrin)
Glyma.08G230500	Pathogenesisrelated protein Bet v I family (Bet_v_1)
Glyma.05G216000	
Glyma.03G194000	AN1TYPE ZINC FINGER PROTEIN
Glyma.01G228700	Naringeninchalcone synthase / Flavonone synthase
Glyma.07G136800	EXPRESSED PROTEIN
Glyma.15G268200	
Glyma.07G076600	BON1ASSOCIATED PROTEIN 1
Glyma.10G013300	CAMPRESPONSE ELEMENT BINDING PROTEIN
Glyma.07G212400	AP2 domain (AP2)
Glyma.01G024000	MITOCHONDRIAL UNCOUPLING PROTEIN 4RELATED
Glyma.12G087200	FAMILY NOT NAMED // CYTOCHROME P450 94C1
Glyma.16G037900	
Glyma.08G153700	
Glyma.20G183800	
Glyma.15G263600	PREMRNA PROCESSING PROTEIN PRP39
Glyma.14G078600	Hydroperoxide dehydratase / Hydroperoxide isomerase
Glyma.17G186600	elongation factor 1alpha (EEF1A)
Glyma.14G212200	WDSAM1 PROTEIN // UBOX DOMAIN
Glyma.15G272300	ZINC FINGER A20 AND AN1 DOMAINCONTAINING STRESS
Glyma.18G029300	Hs1pro1 protein C
Glyma.01G204400	PROTEIN TIFY 10ARELATED
Glyma.05G079700	CALMODULIN1
Glyma.12G183800	AN1TYPE ZINC FINGER PROTEIN
Glyma.10G251900	ubiquitin B (UBB) // ubiquitin C (UBC)
Glyma.05G047100	EFHAND CALCIUM
Glyma.11G185700	CYTOCHROME P450 94C1
Glyma.01G239600	2hydroxyisoflavanone dehydratase (HID)
Glyma.13G279900	NAC DOMAINCONTAINING PROTEIN 19
Glyma.07G151300	Delta(12)fatty acid dehydrogenase / Linoleate Delta
Glyma.15G115300	CYSTATIN FAMILY MEMBER
Glyma.11G096700	EXPANSINLIKE A1
Glyma.17G246500	Hydroperoxide dehydratase / Hydroperoxide isomerase
Glyma.13G236500	AP2 domain (AP2)
Glyma.11G228100	Hs1pro1 protein C

Glyma.03G131900	FAMILY NOT NAMED // PROTEIN EARLY RESPONSIVE TO DEHYDRATION 15
Glyma.16G084300	FAMILY NOT NAMED // AUXININDUCED IN ROOT CULTURES PROTEIN 12
Glyma.13G176400	UBIQUITIN // POLYUBIQUITIN 3
Glyma.07G023300	WRKY TRANSCRIPTION FACTOR 40RELATED
Glyma.06G157400	FAMILY NOT NAMED // GRAB1LIKE PROTEIN
Glyma.02G039700	CLASSICAL ARABINOGALACTAN PROTEIN 10RELATED
Glyma.13G138600	Ubiquitin and ubiquitinlike proteins
Glyma.03G202600	E3 UBIQUITINPROTEIN LIGASE PUB22
Glyma.17G236200	ZINC FINGER PROTEIN AZF3RELATED
Glyma.04G208300	FAMILY NOT NAMED // GRAB1LIKE PROTEIN
Glyma.15G179600	PROTEIN TIFY 10ARELATED
Glyma.06G045400	C2H2type zinc finger (zf
Glyma.05G049900	FAMILY NOT NAMED // DEHYDRATIONRESPONSIVE ELEMENT
Glyma.13G043900	FAMILY NOT NAMED // PHI1
Glyma.11G117300	RNA recognintion motif
Glyma.10G055300	HEAVYMETAL
Glyma.02G040700	MITOCHONDRIAL UNCOUPLING PROTEIN 4RELATED
Glyma.16G039800	
Glyma.15G132800	F22M8.11 PROTEINRELATED
Glyma.10G214500	Proteinsynthesizing GTPase / Peptide
Glyma.13G338400	RING ZINC FINGER PROTEIN // SUBFAMILY NOT NAMED
Glyma.11G077400	DNAJ HOMOLOG SUBFAMILY C MEMBER
Glyma.19G044900	ALLENE OXIDE CYCLASE 1, CHLOROPLASTICRELATED
Glyma.07G190100	
Glyma.15G152900	C2 domain (C2)
Glyma.04G063200	
Glyma.08G218600	WRKY TRANSCRIPTION FACTOR 40RELATED
Glyma.09G071600	PROTEIN TIFY 10ARELATED
Glyma.11G137300	RING ZINC FINGER PROTEIN
Glyma.15G115600	FAMILY NOT NAMED // AUXININDUCED IN ROOT CULTURES PROTEIN 12
Glyma.17G187600	
Glyma.10G256400	CYSTM1 FAMILY PROTEIN ARELATED
Glyma.04G020700	
Glyma.16G109900	
Glyma.11G180500	CCR4ASSOCIATED FACTOR 1 HOMOLOG 11
Glyma.14G041700	LATE EMBRYOGENESIS ABUNDANT HYDROXYPROLINERICH GLYCOPROTEIN
Glyma.15G209300	Leucine Rich Repeat (LRR_1) // Leucine rich repeat Nterminal domain (LRRNT_2) // Leucine rich repeat (LRR_8)
Glyma.12G117000	AP2 domain (AP2)
Glyma.01G030300	Protein of unknown function (DUF1645) (DUF1645)
Glyma.03G236300	RESPIRATORY BURST OXIDASE HOMOLOG PROTEIN B
Glyma.20G124200	Domain of unknown function (DUF4228) (DUF4228)
Glyma.12G073100	LASCORBATE PEROXIDASE 2, CYTOSOLIC
Glyma.02G035200	Protein of unknown function (DUF1645) (DUF1645)
Glyma.14G084700	ETHYLENERESPONSIVE TRANSCRIPTION FACTOR ERF008
Glyma.08G171400	EXPRESSED PROTEIN

Glyma.10G010300	ATMYB2
Glyma.16G010000	PROTEIN TIFY 10ARELATED
Glyma.14G088300	ZINC FINGER PROTEIN AZF3RELATED
Glyma.06G017100	SWI/SNFRELATED CHROMATIN BINDING PROTEIN // HIGH MOBILITY GROUP B PROTEIN 4
Glyma.12G238400	ZINC FINGER A20 AND AN1 DOMAINCONTAINING STRESS
Glyma.13G370000	Protein kinase domain (Pkinase) // NAF domain (NAF) // Protein tyrosine kinase (Pkinase_Tyr)
Glyma.02G195900	E3 UBIQUITINPROTEIN LIGASE PUB22
Glyma.03G207500	large subunit ribosomal protein L21e (RPL21e, RPL21)
Glyma.13G204100	ZINC FINGER A20 AND AN1 DOMAINCONTAINING STRESS
Glyma.13G212500	Prolinerich nuclear receptor coactivator (PNRC)
Glyma.13G219100	PROTEIN TIFY 5ARELATED
Glyma.15G041300	
Glyma.15G202600	GB
Glyma.17G030700	FAMILY NOT NAMED // COBRALIKE PROTEIN 7
Glyma.06G036300	serpin B (SERPINB)
Glyma.02G008500	Nonspecific serine/threonine protein kinase / Threonine
Glyma.15G055800	
Glyma.04G008900	TRANSCRIPTION FACTOR GATA GATA BINDING FACTOR // GATA TRANSCRIPTION FACTOR 14RELATED
Glyma.13G226600	
Glyma.03G138000	SERINE/THREONINEPROTEIN KINASE // CALCIUM
Glyma.06G044600	FAMILY NOT NAMED // 3OXO
Glyma.08G189500	linoleate 9Slipoxygenase (LOX1_5)
Glyma.13G269600	TUBBYRELATED
Glyma.07G005600	Uncharacterized conserved protein
Glyma.07G235900	MYBLIKE DNA
Glyma.06G101500	
Glyma.06G295400	
Glyma.13G301200	Woundinduced protein (DUF3774)
Glyma.05G082200	Leucinerich repeat (LRR) protein associated with apoptosis in muscle tissue // Apoptotic ATPase
Glyma.20G146100	FRIGIDALIKE PROTEIN 4A
Glyma.06G138100	FAMILY NOT NAMED // F11O4.3RELATED
Glyma.05G077100	CHLORIDE CHANNEL // CHLORIDE CHANNEL PROTEIN CLCA
Glyma.01G167300	large subunit ribosomal protein L8e (RPL8e, RPL8)
Glyma.02G106100	PEROXISOMAL ADENINE NUCLEOTIDE CARRIER 1RELATED
Glyma.13G055400	protein SPIRAL1 and related proteins (SPR1)
Glyma.07G066500	FAMILY NOT NAMED // BYPASS1RELATED PROTEIN
Glyma.10G122300	BETA EXPANSIN 6RELATED
Glyma.13G165200	small subunit ribosomal protein S11e (RPS11e, RPS11)
Glyma.08G254500	Phosphogluconate dehydrogenase (NADP(+)dependent, decarboxylating) / Phosphogluconic acid dehydrogenase
Glyma.12G110100	
